# Psoriasis, Atopic Dermatitis, Acne, Inverse Psoriasis and Hidradenitis: A Case Report of Multiple Disease in a Same Young Patient

**DOI:** 10.1002/ccr3.72354

**Published:** 2026-06-23

**Authors:** Emanuele Amore, Federica Trovato, Giovanni Pellacani, Annunziata Dattola, Antonio Giovanni Richetta

**Affiliations:** ^1^ Dermatology Clinic, Department of Clinical Internal Anesthesiological and Cardiovascular Sciences, Sapienza University Rome Italy; ^2^ Dermatology Unit, IDI‐IRCCS Istituto Dermopatico Dell'immacolata Rome Italy

**Keywords:** acitretin, acne, atopic dermatitis, HS, psoriasis, quality of life

## Abstract

We report a case of a 17‐year‐old girl diagnosed with several severe dermatological conditions: atopic dermatitis (AD), inverse and scalp psoriasis, pustular acne, and hidradenitis suppurativa (HS). We consider this case of particular interest because it is therapeutically challenging and because of its psychological impact on the patient's quality of life. We believe that this case may be useful in understanding the management of complex dermatoses as inescapable from the overall assessment of the inflammatory process, which impacts at different levels (skin, cardiovascular, central nervous system, and psyche).

## Key Clinical Message

This case highlights the therapeutic challenges of complex dermatoses in adolescents, emphasizing the need for adaptive treatment strategies. Despite the failure of multiple biologic therapies, acitretin—a retinoid with broad‐spectrum effects—proved effective, suggesting potential shared pathways in autoimmune and autoinflammatory skin diseases. The case underscores the profound psychological burden of chronic dermatologic conditions, highlighting the importance of a multidisciplinary approach to treatment.

## 1 Introduction

The cutaneous immune‐mediated inflammatory diseases (IMIDs) [1] represent diseases with increased incidence globally, influenced by both environmental changes and genetic predisposition [2, 3]. They represent inflammatory conditions belonging to two distinct opposing pathophysiological groups: autoinflammatory and autoimmune. They represent two distinct disorders involving the immune system with different mechanisms. In autoinflammatory diseases, there is a dysregulation of the innate immune system with an overactive response and inflammation, while autoimmune conditions are characterized by an adaptive immune response targeting self‐antigens. In summary, while both autoimmunity and autoinflammatory diseases involve the immune system, their difference lies in the mechanisms of immune activation, the nature of inflammation, and the predominant involvement of either the adaptive or innate immune components.

Acne is a chronic inflammatory disease of the pilosebaceous unit, influenced by different triggers. There is an increased sebum production, follicular hyperkeratosis, and perifollicular inflammation. The condition is regulated by intrinsic factors (such as Cutibacterium acnes, androgens and neuroendocrine elements) and extrinsic factors (including dietary factors, mechanical friction) [4]. Inflammation, in particular, is now recognized as a key factor in all stages of acne lesion development [5].

Hidradenitis suppurativa (HS) is a chronic skin condition characterized by inflammation and follicular occlusion. It affects areas such as the axillae, perineum, and inframammary regions [6]. The etiology of HS is characterized by multifactorial determinants, necessitating a comprehensive and multimodal therapeutic approach. The intricate pathogenesis of HS remains incompletely understood, exhibiting a complex interplay of contributing factors. Autoinflammation emerges as the principal driver in the genesis of the disease, characterized by dysregulated inflammasome activation and subsequent heightened production of inflammatory cytokines. Genetic factors and the mix of microorganisms on the skin also play a role in making this inflammation chronic and causing the formation of skin lesions [7].

Psoriasis is a skin condition caused by a combination of genetic factors and environmental triggers. The immune system plays a key role in psoriasis, as certain immune cells, called T‐helper 17 cells [8], become overactive. These cells produce substances called cytokines, which stimulate the rapid growth of skin cells and cause inflammation [9]. Both genetic predisposition and environmental factors, like infections, can trigger or worsen psoriasis. For instance, streptococcal infections have been linked to flare‐ups of the condition [10].

Atopic dermatitis (AD) is a complex and multifactorial disease, with recent research highlighting the role of type 2 cytokines, particularly IL‐4 and IL‐13, in atopic inflammation [11]. Furthermore, the disease's molecular mechanisms involve genetic disorders, epidermal barrier defects [12], immune response alterations, and skin microbial balance disruption [13]. Environmental factors, including irritants and cutaneous infections, can trigger or exacerbate AD, with the skin being particularly susceptible to microbial infections [14].

## 2 Case Report

### 2.1 Case History

We present a unique case of a young female patient, 17 years old, that encompasses different dermatologic conditions of severe grade: AD, inverse and scalp psoriasis, pustular acne, and HS. The personal and family history was negative for metabolic diseases, the results of laboratory tests are summarized in Table 1. The patient presented at our dermatology department with a 2‐year history of dermatological conditions that manifested one after the other. She had undergone diagnostic biopsies at another hospital, with findings of psoriasiform dermatitis and amicrobial pustulosis of the folds. The patient was unsuccessfully treated with prednisone 25 mg per os 1cp/die and cyclosporine per os 100 mg/bid, both discontinued after 3 months due to ineffectiveness and serious side effects (e.g., myalgia, malaise lipotimic crisis). After worsening of HS with a relapsing–remitting trend, dapsone 100 mg/day was started, with partial improvement. After 12 months, clinical evaluation (CE) showed worsening of the overall clinical picture and others colleagues decided to start biological treatment with adalimumab, in addition to dapsone and metrotrexate for 12 weeks. Due to the worsening of the psoriatic and hidradenitis lesions, they decided to switch to secukinumab, an anti‐IL‐17A monoclonal antibody approved for the treatment of moderate‐to‐severe psoriasis and hidradenitis, with potential use in minors. Secukinumab was administered at the standard dose of 300 mg s.c., with initial dosing at weeks 0, 1, 2, 3, and 4, followed by maintenance dosing of 300 mg every 4 weeks. After 12 weeks, there was no clinical response and treatment was stopped. The same hospital decided to offer dupilumab therapy, but the patient did not start treatment. Also, a genetic study performed on the whole exome showed no alterations in the CARD14 and IL36RN genes, that we know are associated with a rare and severe type of psoriasis and pustular psoriasis. During her journey, the patient reported feelings of frustration related to the illness, so she started psychotherapy. She reported that some days she could not look at herself in the mirror and that the chronic pain she was experiencing was affecting her quality of life and school performance. She also reported episodes of self‐harm. After a psychiatric consultation, she was started on duloxetine and amitriptyline. She was diagnosed with an anxious state and depressed mood.

**TABLE 1:** Laboratory findings and BMI (pre‐treatment baseline).

Test name	Result	Normal range
Hemoglobin (Hb)	13.2 g/dL	12.0–15.5 g/dL
WBC count	7.8 x 10^9^/L	4.0–11.0 x 10^9^/L
Neutrophils	62%	50%–70%
Lymphocytes	28%	20%–40%
Platelets	250 x 10^9^/L	150–400 x 10^9^/L
CRP	7.5 mg/L	< 5 mg/L
ESR	22 mm/h	< 20 mm/h
ALT	22 U/L	< 35 U/L
AST	18 U/L	< 35 U/L
Creatinine	0.7 mg/dL	0.5–1.1 mg/dL
BUN	12 mg/dL	7–20 mg/dL
Fasting glucose	88 mg/dL	70–99 mg/dL
Insulin	9.5 μU/mL	2.0–25.0 μU/mL
Total cholesterol	190 mg/dL	< 200 mg/dL
LDL	120 mg/dL	< 100 mg/dL
HDL	45 mg/dL	> 40 mg/dL
Triglycerides	150 mg/dL	< 150 mg/dL
TSH	2.5 μIU/mL	0.5–4.5 μIU/mL
Free T4	1.1 ng/dL	0.8–1.8 ng/dL
HOMA‐IR	2.1	< 2.6
ANA	Negative	Negative
Rheumatoid factor	Negative	Negative
HLA‐C06	Negative	Negative
HLA‐C07	Positive	Positive
BMI	22.0 kg/m^2^	18.5–24.9 kg/m^2^

### 2.2 Examination

**FIGURE 1:**
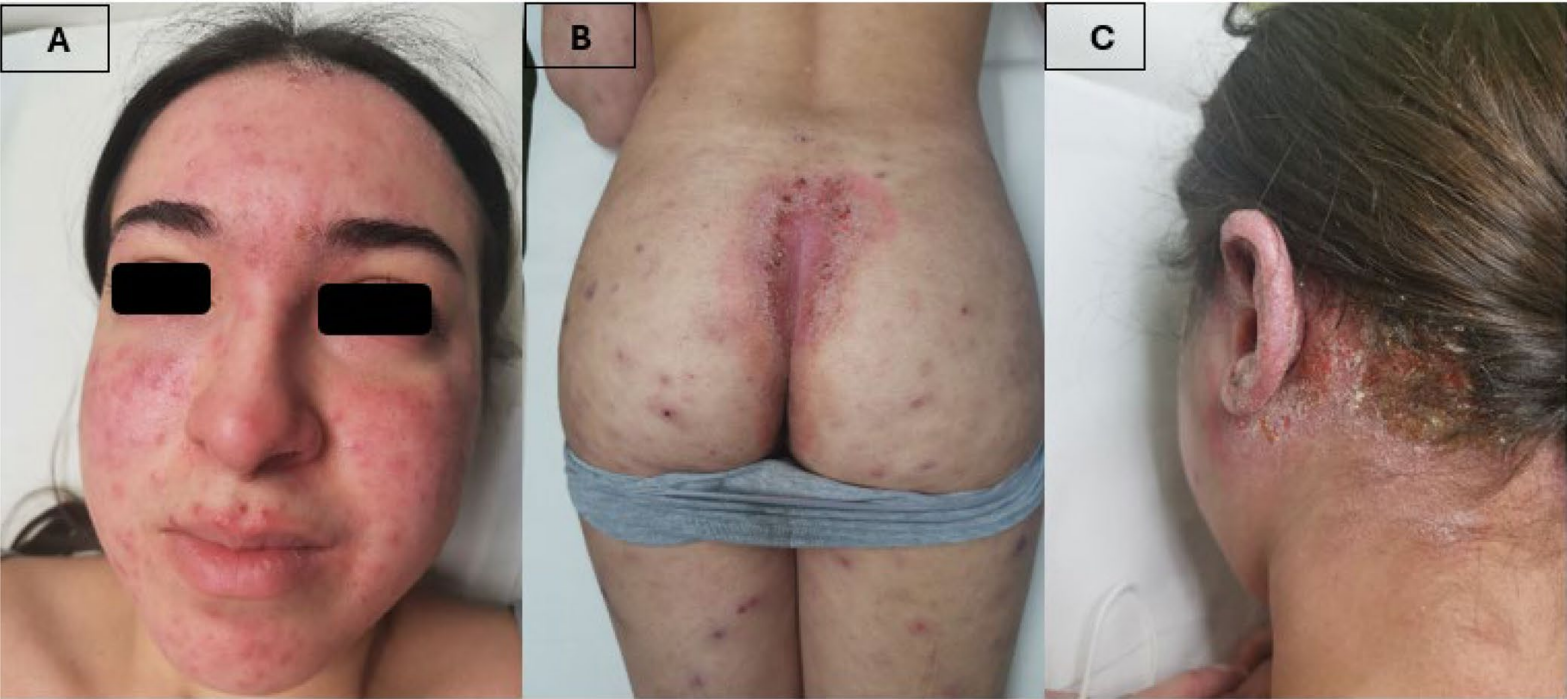
Evaluation at T0. (A) Inflamed, erythematous papulo pustules in the malar, nasal, chin, and frontal areas, suggesting acne. (B) Scaly plaques around the external genitalia and perineum, suggesting psoriasis. (C) Scaly plaques on the skin and scalp, suggesting psoriasis.

In January 2023 the patient self‐referred to our Department of Dermatology in Rome at La Sapienza University. Examination revealed the presence of red, scaly plaques on the skin and scalp, around the external genitalia and perineum, suggestive of psoriasis; inflamed, erythematous papulo‐pustules in the malar, nasal, chin, and frontal areas, suggestive of acne; erythematous, itchy, lichenified areas at the popliteal and elbow folds, suggestive of AD, at this time we also found post inflammatory lesions and scars that followed hidradenitis (Figure 1).

### 2.3 Investigations and Treatment

After the failure of two monoclonal antibodies, we found the therapeutic decision very challenging. There were no viable biologic therapies for psoriasis and HS in patients under 18 years of age. The turning point came when we opted for treatment with acitretin 25 mg/die, which is generally not used in childbearing women because of its ability to deposit in adipose tissue and its teratogenic effects.

### 2.4 Outcome and Follow‐Up

**FIGURE 2:**
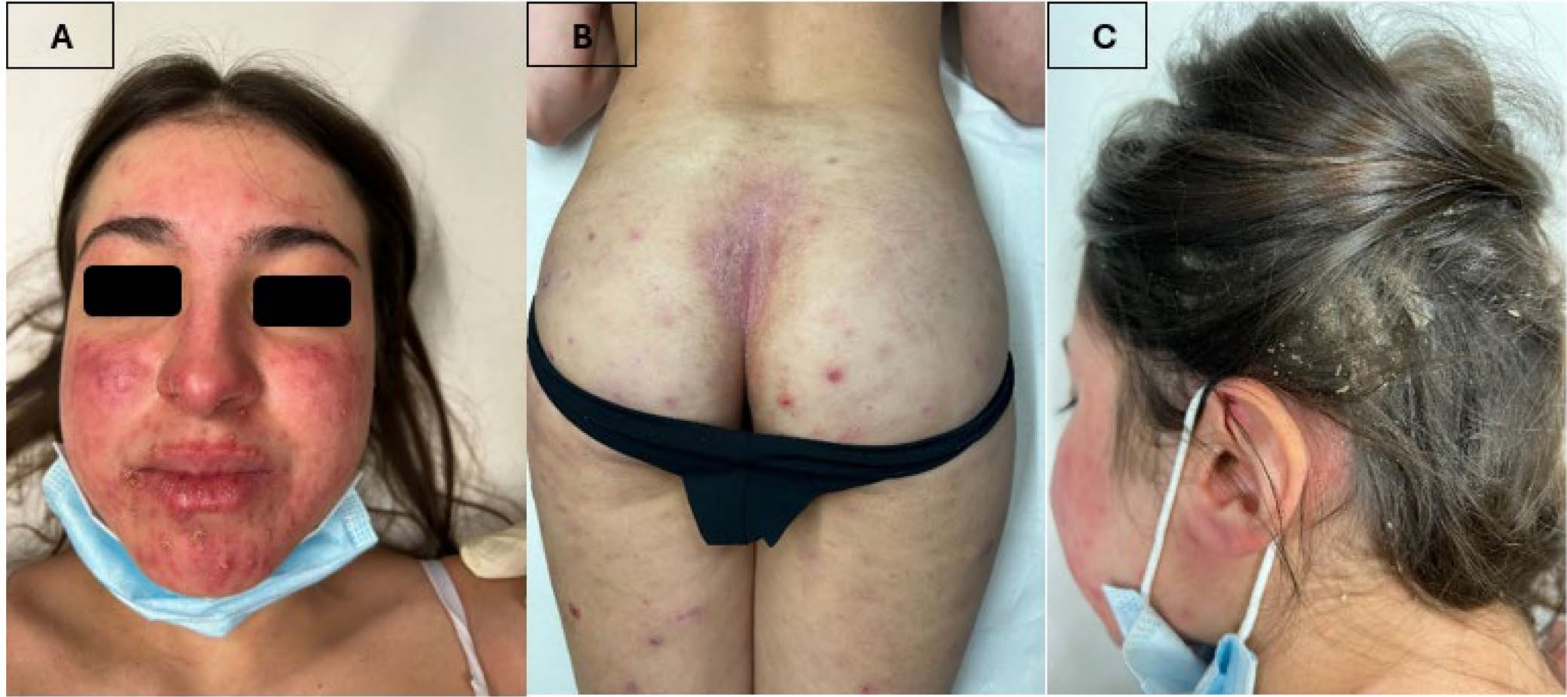
10 weeks follow‐up visit. (A) Significantly less pustules and diffuse erythema. (B) The lesions showed considerable improvement. (C) Lesions in the retro‐auricular region and scalp are improving.

**FIGURE 3:**
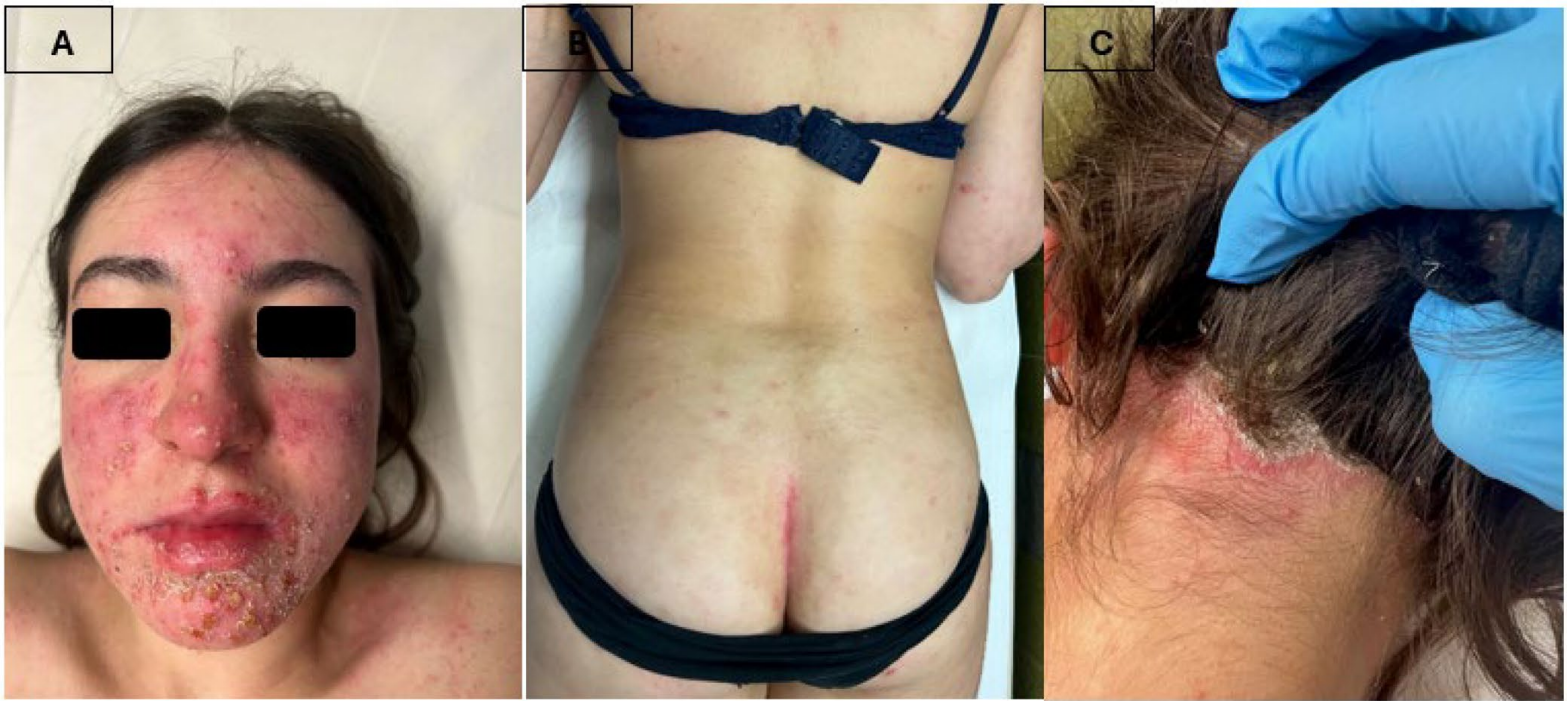
12 weeks follow‐up visit. (A) The facial erythema slightly improved, but the pustules remained. (B) Almost complete resolution of genital and intergluteal psoriatic lesions. (C) Improvement but persistence of psoriatic lesions on the scalp.

**FIGURE 4:**
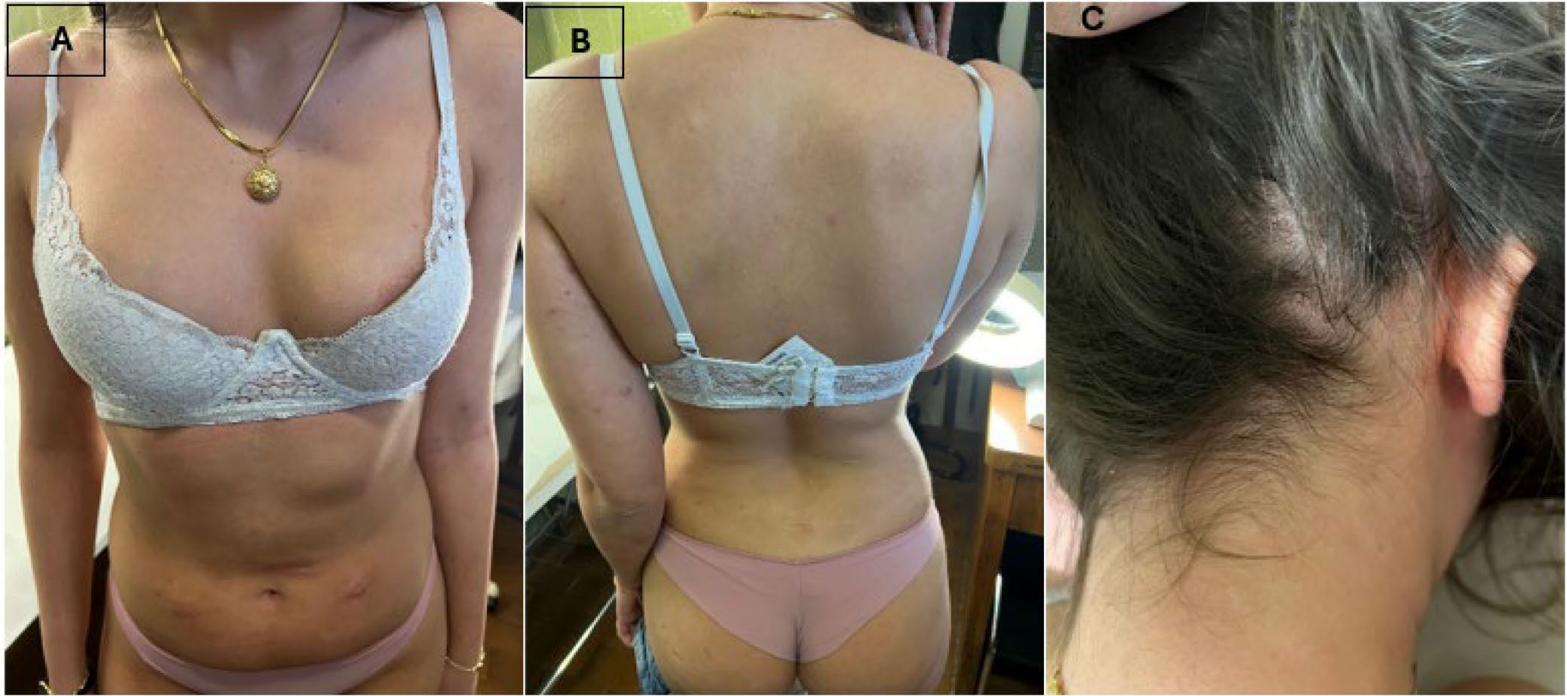
20 weeks follow‐up visit. Complete resolution of lesions on the chest (A), dorsal area (B) and scalp (C).

**FIGURE 5:**
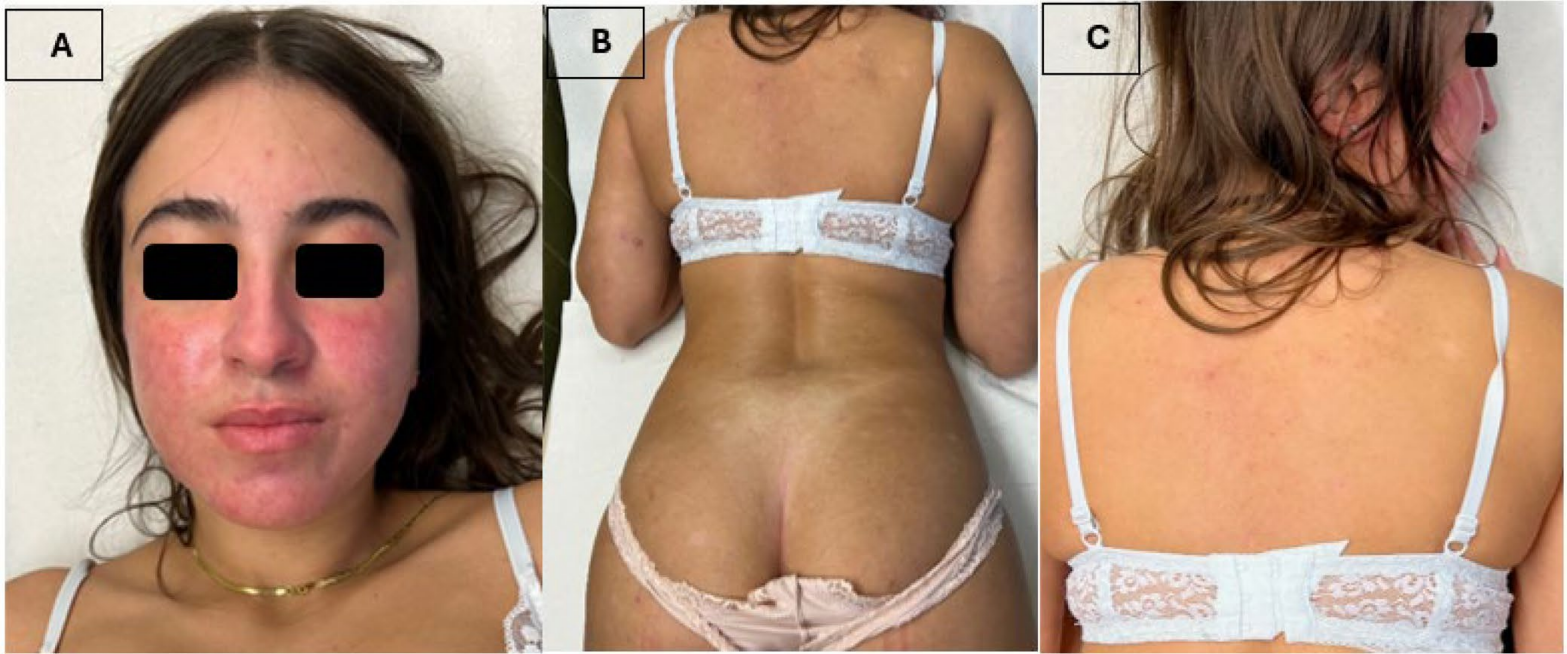
50 weeks follow‐up visit. Discontinuation of Acitretin. Resolution of all conditions (B, C) except acne, which remains active (A).

**FIGURE 6:**
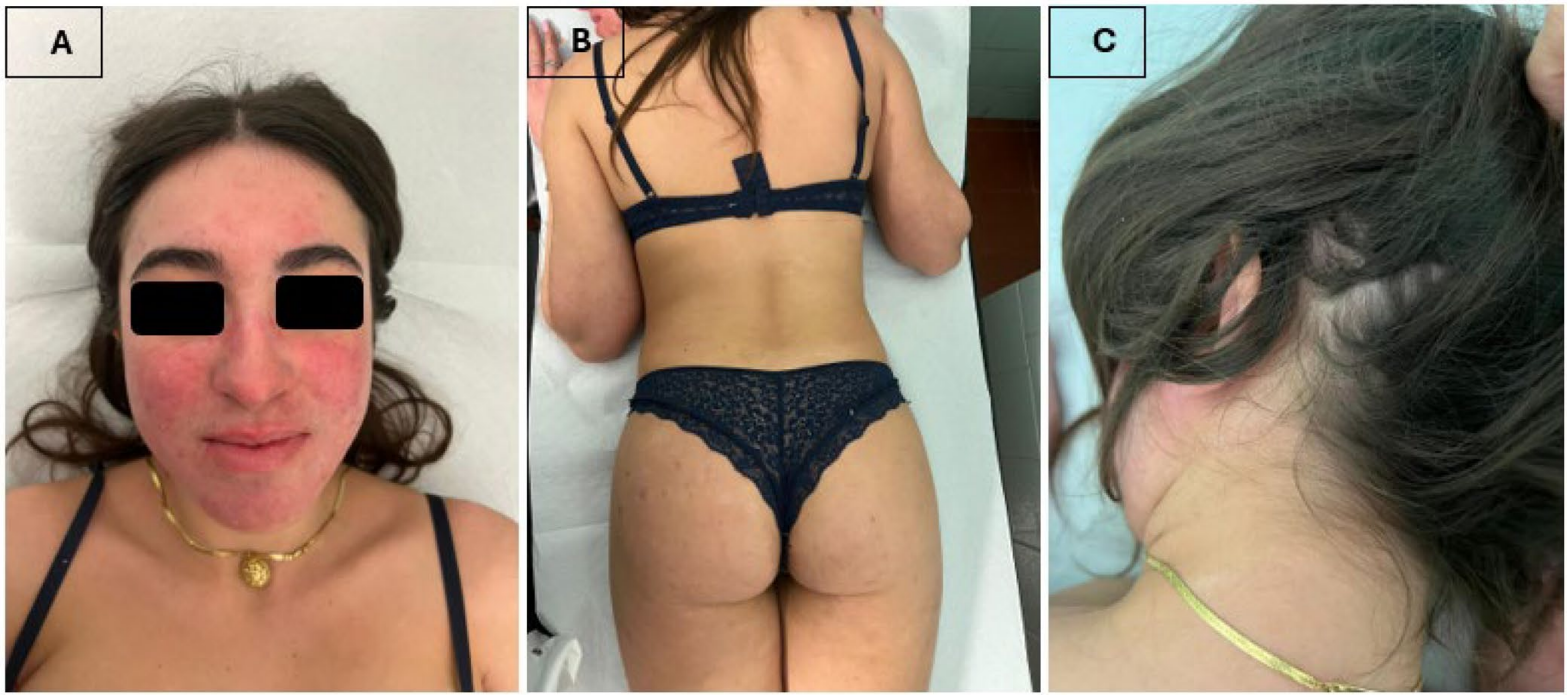
52 weeks follow‐up visit. Improvement of acne lesions after 2 weeks on Isotretinoin (A). Absence of new lesions of psoriasis, atopic dermatitis, nor hidradenitis (B, C).

After 2 weeks, CE showed a slight improvement. We followed the patient every 2 weeks and after 8 weeks we decided to increase the dose of acitretin to 30 mg/die and to do a genetic test to look for HLA C06, which we know is associated with an excellent response in psoriasis‐positive patients. HLA C06 was negative and the patient was positive for HLAC07. At the next visit CE showed marked improvement of the cutaneous lesion, but new lesions suggestive of AD appeared at the popliteal fossa, antero‐cubital fossa, and left thigh. The facial area showed diffuse erythema, probably ascribed to therapy, with marked reduction of pustules; on the scalp and retro‐auricular regions there was improvement of lesions (Figure 2). At 12 weeks mild improvement of the facial erythema was noted, but the pustules persisted. We decided to temporarily discontinue acitretin 30 mg/day therapy and add lymecycline 300 mg/day for 12 weeks. During acne breakouts, she required a medical certificate to wear a surgical mask during school classes due to the significant psychological impact. She felt uncomfortable showing her face to classmates (Figure 3). At week 16, CE showed a decrease in atopic and psoriatic features, and the dosage of acitretin was reduced to 20 mg/day. After further clinical improvement at 4 weeks, acitretin was decreased to 10 mg/die, with maintaining clinical well‐being obtained 24 weeks after initiation of therapy. Mild worsening of scalp psoriasis was successfully treated with topical application of clobetasol propionate for 28 days (Figure 4). The patient has now reached 1 year of follow‐up with marked improvement and resolution of all conditions except acne, which remains active, so we have decided to discontinue acitretin (Figure 5) and start treatment with isotretinoin per os [15] (Figure 6). Patient has also discontinued pharmacological psychiatric therapy and is currently continuing psychotherapy, with significant improvement in her mental health.

## 3 Discussion

Complex dermatoses represent a therapeutically challenging field. Complexity increases if there is a concurrence of different diagnoses, especially if the patient is a minor. In this multisided case, many last‐generation therapeutic chances (Adalimumab, Secukinumab) have apparently failed in managing the disease [16]. Surprisingly, a “vintage” drug such as Acitretin has been successful in controlling the disease. Our hypothesis is based on Acitretin's broader‐spectrum mechanism of action than monoclonal antibodies.

Acitretin is a retinoid medication; it works by binding to nuclear receptors in the skin cells, particularly retinoic acid receptors, which regulate gene transcription. This binding leads to changes in gene expression, promoting normal growth and differentiation of skin cells while reducing inflammation. It regulates cell differentiation, proliferation and apoptosis; activates the nuclear retinoic acid receptor, causing neutrophils' inhibition [17]. Lately, Jie Tu et al. [18] explained a new role of acitretin in the inhibition of IL‐36 expression induced by IL‐17. Acitretin suppresses IL‐36β and IL‐36γ expression in keratinocytes significantly in mouse models. Acitretin also down‐regulates IκBζ, consequently inhibiting IL‐36 expression induced by IL‐17A stimulation in keratinocytes. This could be a potential mechanism underlying the remarkable and rapid therapeutic efficacy of acitretin on pustular acne and psoriasis [18, 19, 20].

An important aspect of IMIDs concerns the psychosocial impact they have on the patient's quality of life, especially in adolescence. Several studies have demonstrated a notable correlation between the severity of acne and levels of anxiety, depression, and quality of life [21]. Numerous researches, including works by Gieler [22] and Zip [23], have highlighted the profound psychosocial repercussions of acne, ranging from feelings of depression and social withdrawal to even contemplating suicide. Of particular note is the vulnerability of adolescents and young adults to the psychosocial aspect of acne, exacerbated by norms of beauty and the pressures of acceptance [24]. Tasoula et al. have assessed how acne vulgaris affects social and physiological functions in proportion to the severity of acne [25]. Psoriasis greatly affects patients' overall well‐being, including their physical, emotional, financial, and psychological states. Skin pain and discomfort are particularly linked with a lower quality of life for individuals with psoriasis [26]. Zięciak et al. [27] investigated the relationship between feelings of stigmatization and symptoms of depression in psoriasis patients. They found that there is a significant association between stigmatization and depression symptoms among individuals with psoriasis. This suggests that addressing stigmatization may be important in managing depression in psoriasis patients [27].

Evidence in the literature shows that the association between dermatosis and psychosocial impairment is actually linked to the pathophysiological mechanisms of inflammatory diseases. Stress, anxiety, and other psychological factors can exacerbate dermatoses through the release of vasoactive neuropeptides and other chemical mediators [28]. Inflammatory skin diseases can also lead to increased psychological stress, creating a cyclical relationship [29]. Atopic dermatitis, a chronic inflammatory disease, is particularly associated with comorbidities such as depression and anxiety [30]. A recent study conducted by Zhang explained how stress can impact skin disease by studying neuroendocrine‐immune interaction. Stress impacts the immune system through the HPA axis, sympathetic nervous system, and neuropeptides, affecting skin diseases [31].

## 4 Conclusions

We strongly believe that this patient's unique clinical journey is valuable in highlighting the importance of adaptive therapeutic strategies in the context of complex dermatoses. It also suggests the possibility of shared etiopathogenetic pathways among different dermatoses, as previously suggested by Lindor et al. and Marzano et al. in their definition of the PAPA and PAPASH syndromes [32, 33]. Despite advances in the therapeutic field, there is still little attention given to the psychosocial impact of chronic dermatoses. Our clinical case paves the way to the hypothesis of a common basis for dermatoses of different etiopathogenesis, autoimmune and autoinflammatory, and aims to focus attention on the topic of psychological impairment in skin diseases.

## Author Contributions

**Emanuele Amore:** conceptualization, writing – original draft, writing – review and editing. **Federica Trovato:** conceptualization, data curation, formal analysis, supervision, validation, visualization, writing – original draft, writing – review and editing. **Giovanni Pellacani:** data curation, formal analysis, funding acquisition, supervision. **Annunziata Dattola:** validation, visualization, writing – original draft, writing – review and editing. **Antonio Giovanni Richetta:** validation, visualization.

## Funding

The authors have nothing to report.

## Consent

Written informed consent was obtained from the patient for the publication of this case report and any accompanying clinical images. The patient consented to the use of their information in this report, and ethical approval was granted by the Sapienza Ethics Committee.

## Data Availability

Data sharing is not applicable to this article as no new data were created or analyzed in this study.
